# Molecularly Targeted Therapy for Neuroblastoma

**DOI:** 10.3390/children5100142

**Published:** 2018-10-15

**Authors:** Emily G. Greengard

**Affiliations:** The University of Minnesota Masonic Children’s Hospital/Masonic Cancer Center, Riverside Avenue, Minneapolis, MN 55454, USA; emilyg@umn.edu

**Keywords:** neuroblastoma, molecular guided therapy, targeted agents, precision medicine

## Abstract

Neuroblastoma is the most common extra-cranial solid tumor encountered in childhood and accounts for 15% of pediatric cancer-related deaths. Although there has been significant improvement in the outcomes for patients with high-risk disease, the therapy needed to achieve a cure is quite toxic and for those that do experience a disease recurrence, the prognosis is very dismal. Given this, there is a tremendous need for novel therapies for children with high-risk neuroblastoma and the molecular discoveries over recent years provide hope for developing new, less toxic, and potentially more efficacious treatments. Here I discuss many of the molecular aberrations identified thus far in neuroblastoma, as well as the agents in development to target these changes. The progress made in both the preclinical arena and in early phase drug development provide much promise for the future of precision medicine in neuroblastoma.

## 1. Introduction

Over recent decades, significant progress has been made in understanding the diverse clinical behavior of neuroblastoma and in the ability to risk stratify a patient at the time of diagnosis. The biology of a patient’s tumor plays a large role in determining the risk group for the patient and considerable efforts in the field of “omics” have accelerated our understanding of neuroblastoma biology. With this greater understanding there is tremendous momentum to translate this knowledge not only into improved prognostic stratification, but also more personalized therapy or precision medicine. Many treatment protocols for relapsed and refractory neuroblastoma are based on genomic profiling of the tumors and resultant molecularly guided therapies. In addition, the most novel upfront treatment protocols are attempting to identify biomarkers and molecular aberrations in the tumors in order to incorporate novel agents to target those discoveries. The molecularly guided therapies that have been investigated and continue to be developed are discussed here (Table A1).

## 2. The Role of MYCN in Neuroblastoma

MYCN amplification was determined to be a strong prognostic factor for survival in neuroblastoma in the late 1980s, and today remains one of the most important validated biomarkers [1]. Since the correlation between MYCN status and prognosis was discovered, multiple attempts to develop a therapy to target MYCN and impair its function have been undertaken. Targeting MYCN has proven to be challenging due to a lack of appropriate surfaces on its DNA binding domain to which drugs can bind. It appears as though the most effective approach for inhibiting MYCN or controlling its regulation is through indirect targeting [2,3]. The following agents continue to be studied in children with neuroblastoma due to their role in regulating MYCN.

### 2.1. Aurora A and Aurora B Inhibitors

The utility of inhibiting Aurora A kinase for the treatment of neuroblastoma has been extensively investigated and there continues to be an ongoing interest in inhibiting this kinase. Both Aurora A (AURKA) and Aurora B (AURKB) are crucial regulators of the cell cycle. AURKA is critical for mitotic spindle assembly and stability as well as regulation of centrosome and kinetochore formation [4]. AURKA also plays a crucial role in the success of MYCN. The half-life of MYCN is only approximately 30 min; however, AURKA stabilizes MYCN through a direct protein-protein interaction, making it less degradable by the proteasome. AURKB has been confirmed as a direct transcriptional target of MYCN. Expression of AURKA and/or B have both been correlated with poor prognosis in neuroblastoma and are candidates for targeting with specific inhibitors [5]. MLN8237 (alisertib) was studied in pre-clinical models and subsequently in a phase 1 clinical trial for children with relapsed/refractory solid tumors. Both once daily and twice daily dosing were studied; however, dose-limiting toxicities (DLTs) of mucositis and myelosuppression were seen with twice daily dosing. The recommended phase 2 dose (RP2D) was found to be 80 mg/m^2^/day once daily for 7 days every 21 days [6]. As a single agent the response rate was low however, pre-clinical data suggest that MLN8237 synergizes with irinotecan and temozolomide and thus this combination was subsequently studied in children with relapsed or refractory neuroblastoma. The overall response rate in this combination phase 1 trial of 31.8% was encouraging; however, there are currently no ongoing trials investigating the use of this agent in neuroblastoma [7]. Perhaps a mechanism to provide a greater anti-tumor effect than targeting AURKA alone is targeting both AURKA and AURKB through the use of pan-aurora inhibitors. Indeed, tozasertib (VX680, MK-0457), a pan-aurora inhibitor, was found to have potent activity in a panel of drug-resistant neuroblastoma cell lines [8]. Although this agent has been studied in adult patients with malignancies, it has yet to be studied in pediatrics.

### 2.2. Inhibitors of MYCN/Max Interactions

Although targeting the MYCN/Max interaction is an appealing strategy, poor solubility and short half-lives of the agents currently available have limited their clinical development. MYC requires its partner MAX in order to fully function as a transcriptional activator [9,10]. Two compounds, 10074-G5 and 10058-F4, both block the MYC-MAX interaction [11,12,13]. In both in vitro and in vivo MYCN amplified models, treatment with these agents has been promising, inducing differentiation and apoptosis in cell lines and tumor growth suppression in xenografts. Unfortunately, agents with more potential for clinical use are not yet available or in development.

### 2.3. BET Inhibitors

Indirect targeting of MYCN can also be accomplished by inhibition of the bromodomain and extra-terminal domain (BET) family of proteins. BET proteins are a group of epigenetic modifiers that are important for transcriptional regulation of many genes, including MYCN. Three small molecule inhibitors of BET have been investigated in pre-clinical models and have been shown to downregulate MYCN expression, induce cell cycle arrest and apoptosis in neuroblastoma cells and inhibit tumor growth in mouse models [14,15]. Several BET inhibitors are being explored in adolescents and young adults with malignancies but have not yet begun testing in younger children (NCT02419417, NCT01587703).

### 2.4. Targeting MYCN Downstream Pathways: P53/MDM2

There is significant evidence to suggest that the p53/MDM2/p14^Arf^ pathway may be a rational target in neuroblastoma. Many adult cancers and some pediatric cancers are associated with mutations in TP53. Although this is not the case in neuroblastoma, there is evidence that p53 pathway inactivation often occurs at the time of relapse and likely contributes to chemo resistance. Several mechanisms for this inactivation have been proposed including MDM2 gene amplification or increased MDM2 expression mediated by MYCN [16,17,18]. In addition, several chromosomal aberrations commonly seen in neuroblastoma, including gain of 17q and LOH of 1p, alter p53 function. Inhibition of MDM2 has been the most extensively studied mechanism of restoring p53 activity in neuroblastoma [19]. The small molecule inhibitors nutilin-3 and MI-219 interact with MDM2 by mimicking the p53 N-terminal region, where MDM2 binds to p53. Preclinical investigation of these MDM2 inhibitors show that the effects depend on the MYCN status of the cells in that MYCN over-expression sensitizes the cells to MDM2 inhibition [20]. Both in vitro and in vivo studies of these agents have been promising as single agents and in combination with agents such a bevacizumab, venetoclax and temsirolimus [21,22,23,24]. The MDM2 inhibitors DS-3032b and AMG-232 are currently being investigated in multiple adult oncology trials; however, pediatric trials have not yet been initiated.

### 2.5. Targeting MYCN Downstream Pathways: ODC1 Inhibitors

The Ornithine decarboxylase 1 (ODC1) gene encodes an enzyme that is the rate limiting step in polyamine synthesis. Polyamines play an important role in neuroblastoma in that they are cationic chaperones that support MYC activities through ionic and covalent mechanisms and are critical to initiating and maintaining the cancer phenotype. Given the interaction of MYC and the polyamine pathway, significant effort has been devoted to understanding the role of polyamines in supporting neuroblastoma initiation and progression [25,26].

Difluoromethylornithine (DFMO) is an irreversible ODC1 inhibitor that is FDA approved for the treatment of Trypanosomiasis. In a transgenic mouse model of neuroblastoma, treatment with DFMO delayed tumor initiation and synergized with chemotherapy to extend the survival of mice with established tumors [25]. Further preclinical studies have demonstrated that combined inhibition of ODC1 and AMD1 using DFMO and SEAM486 prior to tumor initiation profoundly reduced tumor penetrance in TH-MYCN neuroblastoma-prone mice, a transgenic mouse model that harbors mis-expression of the human MYCN gene in neural-crest derived cells [27]. In addition, combined treatment with DFMO and celecoxib resulted in synergistic antitumor activity in models with MYCN amplification, ALK mutation and TP53 mutations [28]. DFMO has been studied in children with neuroblastoma and doses up to 3 g/m^2^/day were tolerable [29]. There are ongoing studies of single agent DFMO for the prevention of disease relapse as well as trials combining it with either topotecan and cyclophosphamide (topo/cyclo) and celecoxib or bortezomib for the treatment of relapsed or refractory neuroblastoma. Results are these trials are pending; however, it is likely that combining DFMO with other anti-neoplastic agents will be the optimal approach to the development of this agent in neuroblastoma (NCT01586260, NCT02030964, NCT02139397).

### 2.6. Targeting MYCN Downstream Pathways: PI3K/AKT/mTOR Inhibitors

The PI3K/AKT/mTOR pathway appears to play a role in MYCN stabilization and aberrant activation of the pathway has been demonstrated in neuroblastoma. For these reasons, it has been a focus for the development of targeted therapies. PI3K inhibition in MYCN-driven murine neuroblastoma leads to decreased levels of MYCN protein and tumor regression [30,31]. Numerous inhibitors of the PI3K/AKT/mTOR pathway have been developed; however, the question still remains as to what the optimal target in this pathway is. Temsirolimus, an mTOR inhibitor, was studied in combination with irinotecan and temozolomide through a Children’s Oncology Group (COG) phase 1 trial for children with relapsed or refractory solid tumors. This study defined the RP2D as 35 mg/m2/dose IV weekly when used in combination with irinotecan and temozolomide [32]. The combination was then deemed ineffective when compared to dinutuximab plus irinotecan and temozolomide in a COG Phase 2 trial where only 1 patient (6%) achieved a partial response [33]. It is unlikely that this agent will be further developed for use in neuroblastoma. The dual mTORC1-mTORC2 inhibitor AZD8055 has been evaluated in the pre-clinical realm in both neuroblastoma cell lines and mouse models. The agent inhibited cell growth and induced apoptosis in cell lines and significantly decreased tumor growth in xenografts without causing apparent toxicity [34]. The agent has been extensively studied in adult trials but has yet to be investigated in the pediatric population.

Perhaps a better approach is broader inhibition of the PI3K/AKT/mTOR pathway. The New Approaches to Neuroblastoma Therapy (NANT) consortium has an ongoing phase 1 trial of SF1126, a pan-PI3K/mTOR inhibitor that is a novel RGDS-conjugated LY294002 prodrug. SF1126 has enhanced delivery to the tumor vasculature and tumor itself due to its increased solubility and ability to bind to integrins in the tumor compartment [35]. The goal of the NANT trial is to determine the RP2D in the initial cohort and then to study an expansion cohort of patients whose tumors demonstrate MYCN-amplification or MYC overexpression (NCT02337309). In addition, a phase 1/2 trial of the PI3K inhibitor, copanlisib, is currently ongoing through the COG and includes a cohort for patients with relapsed or refractory neuroblastoma (NCT03458728). Finally, combined targeting of AKT has been proposed as another potentially more successful approach to inhibiting this pathway. A phase 1/1b trial of the AKT inhibitor, perifosine, was completed in children, adolescents and young adults with advanced solid tumors. Twenty-seven patients with relapsed or refractory neuroblastoma were treated in either the phase 1 or phase 1b expansion cohorts. The medication was well tolerated without any DLTs and 33% of patients had a progression free survival for a median of 54 months from study entry (range: 43–74 months) [36].

### 2.7. Disruption of the Core Regulatory Circuity and Transcriptional Interference

Core regulatory circuits (CRCs) are formed by transcription factors assembled into feed-forward autoregulatory loops that establish and maintain cell lineage and identity through extended regulatory networks. Recently, through the use of a genome-scale CRISPR-Cas9 approach, within 147 candidate genes found to be selectively essential to the growth and survival of MYNC-amplified neuroblastoma cell lines, it was discovered that a small group of transcriptional factors are members of the CRC that maintains cell state in MYCN amplified neuroblastoma. Depletion of expression of these genes resulted in suppression of colony formation and induction of apoptosis in MYCN amplified neuroblastoma cell lines. Based on this, it was hypothesized that disrupting the CRC and transcriptional initiation and elongation through BRD4 and CDK7 inhibition may be an effective strategy for treating neuroblastoma. Neuroblastoma cell lines were treated with the combination of the BRD4 inhibitor, JQ1, and theCDK7 inhibitor, THZ1. Single agent treatment with either agent resulted in reduced cell growth however, the combination was found to be synergistic, dramatically reducing cell numbers and inducing apoptosis. This combination was also evaluated in a mouse model and resulted in reduced tumor progression and increased survival compared to either single agent alone. The effects of combined BRD4 and CDK7 inhibition on both CRC and global gene expression were evaluated in neuroblastoma cell lines. In comparison to the top 1% of highest expressed transcripts, CRC mRNA levels rapidly declined by 1 h after treatment [37]. The dual P13K/BRD4 inhibitor, SF1126, is currently being evaluated through the NANT consortium (NCT02337309). There are ongoing trials of CDK7 inhibitors in adult patients with malignancies but none yet in children. In addition, the combination of BRD4 and CDK7 inhibitors has yet to be studied in the clinical realm.

## 3. ALK Inhibitors

Currently, the most druggable target for neuroblastoma is anaplastic lymphoma kinase (ALK). The ALK gene resides on chromosome 2p23.1 and is a receptor tyrosine kinase belonging to the insulin receptor superfamily. ALK is normally expressed in the developing nervous system and is thought to play a role in neuronal differentiation. ALK translocations have been described in many malignancies of adults and children. Although ALK translocations are not typically seen in neuroblastoma, approximately 14% of children with high-risk neuroblastoma have tumors that harbor either ALK mutations or amplification. In this high-risk cohort, the presence of an ALK aberration is associated with an inferior 5-year event-free survival, and the hope is that through the addition of ALK inhibition, survival can be improved. ALK mutations are also implicated in the majority of familial neuroblastoma cases [38].

The most extensively studied ALK inhibitor in neuroblastoma, crizotinib, is a small molecule competitive inhibitor of ALK and MET kinase activity and is FDA approved for use in adult patients with ALK-translocated non-small cell lung cancer (NSCLC). Single agent crizotinib was evaluated in children with relapsed and refractory solid tumors or anaplastic large cell lymphoma through a COG Phase 1/2 trial which determined the RP2D to be 280 mg/m^2^/dose twice daily [39]. This trial had a dedicated phase 2 cohort for individuals with ALK aberrant neuroblastoma. Of the 11 patients enrolled on this cohort, 1 patient had a CR and 2 had SD. Those who achieved either a CR or SD received anywhere from 4 to 39 cycles of therapy, with some continuing on treatment at the time of publication of the phase 1 results [39]. Resistance is a real challenge with crizotinib and there is sufficient preclinical data to suggest that crizotinib synergizes with chemotherapy. Preclinical studies of crizotinib have demonstrated that neuroblastoma with the F1174L mutation, the second most frequent ALK mutation observed in neuroblastoma, is resistant to single agent crizotinib. This resistance can be overcome when crizotinib is combined with a chemotherapy regimen including topotecan and cyclophosphamide (topo/cyclo). This was demonstrated in mouse xenografts harboring the F1174L mutation, in that mice treated with the combination of crizotinib plus topo/cyclo had rapid and sustained tumor regression with improved EFS compared to mice treated with single agent crizotinib or topo/cyclo alone. Complete responses were maintained for an additional 24 weeks after cessation of therapy [40]. A COG phase 1 trial evaluated the safety and toxicity of crizotinib in combination with topo/cyclo in children with relapsed and refractory solid tumors and found the R2PD of crizotinib when combined with chemotherapy to be 215 mg/m^2^/dose twice daily (NCT01606878) [41]. The COG is now conducting a phase 3 clinical trial to determine whether the addition of crizotinib to standard of care therapy for high risk neuroblastoma improves survival for patients whose tumor harbors an ALK aberration (NCT03126916).

The sensitivity of neuroblastoma cells to ALK tyrosine kinase inhibitors depends on the specific mutation site, which ultimately impacts ALK binding to either ATP or the tyrosine kinase inhibitor. Data suggests that some mutations are more sensitive to crizotinib than others and resistance can be overcome either by using very high doses of crizotinib or combining it with chemotherapy as described above [40]. Additionally, it appears that newer generation ALK inhibitors may be more effective against crizotinib resistance mutations. These ALK inhibitors continued to be explored in neuroblastoma and include: ceritinib, ensartinib, entrectinib, lorlatinib and alecintib. Ceritinib is a second-generation small molecule ATP competitive tyrosine kinase inhibitor of ALK and IGF-1 [42,43]. Enzymatic assay studies have demonstrated ceritinib to be 20 times more potent against ALK than crizotinib and preclinical studies in NSCLC have demonstrated antitumor activity against both crizotinib-sensitive and crizotinib-resistant NSCLC. Ceritinib is FDA approved for the treatment of patients with ALK positive metastatic NSCLC who have progressed on or are intolerant of crizotinib [44]. A phase 1 study of ceritinib in children with ALK aberrant malignancies has been completed with results pending (NCT01742286). Despite the increased potency of ceritinib over crizotinib, resistance remains a challenge with single agent ceritinib. One potential approach to combating resistance is through the use of novel-novel combinations. The combination of ceritinib and the CDK4/6 inhibitor, ribociclib, has been studied extensively in preclinical models of ALK mutated neuroblastoma. In the ALK mutated cell lines the combination was synergistic, whereas in xenografts, the combination resulted in complete and sustained tumor regression and prolonged EFS compared to either single agent alone. When investigating the relationship of ALK and CDK4/6, it was found that constitutive ALK signaling drives the activation of CDK4 and CDK6 and that treatment with the combination of ceritinib and ribociclib provides both upstream and downstream receptor tyrosine kinase inhibition [45]. The Children’s Hospital of Philadelphia is studying this combination in their Next Generation Personalized Neuroblastoma Therapy (NEPENTHE) trial (NCT02780128). Ensartinib is also a second generation ALK inhibitor with 10-fold increased potency in blocking ALK phosphorylation over crizotinib. Preclinical models of ALK mutated neuroblastoma have shown promising results when treated with ensartinib and this agent in being studied in children with relapsed and refractory solid tumors with ALK aberrations through the NCI/COG Pediatric Molecular Analysis for Therapy Choice (MATCH) trial (NCT03213652). Lorlatinib (PF-6463922) is a highly potent ALK/ROS1 inhibitor that has substantially increased ALK binding affinity over crizotinib. Lorlatinib was studied in preclinical models of neuroblastoma with impressive results, inducing complete tumor regression in xenografts with and without primary resistance to crizotinib [46]. This encouraging data led to a phase 1 trial of loratinib for patients with ALK aberrant neuroblastoma that is currently ongoing through the NANT consortium (NCT03107988). Finally, alecitinib, a second-generation ALK inhibitor with impressive efficacy and toxicity results when compared head-to-head to crizotinib for NSCLC has also had promising results in preclinical neuroblastoma models [47]. As a single agent, alectinib was effective in inducing apoptosis in cell lines with the ALK F1174L mutation [48]. A trial investigating alecitinib for both adult and pediatric patients with ALK mutated or rearranged malignancies is currently enrolling patients (NCT03194893).

## 4. RAS-MAPK and MEK Inhibitors

The RAS-MAPK pathway has been implicated in a variety of adult and pediatric malignancies and plays as role in cellular growth, survival and differentiation. Although only 3–5% of primary neuroblastomas harbor mutations in the RAS-MAPK pathway, close to 80% of relapsed samples contain mutations in genes implicated in this pathway. The RAS-MAPK pathway activating mutations detected in these relapsed neuroblastoma tumors include mutations in ALK, NF1, BRAF, PTPN11, FGFR1, KRAS, HRAS and NRAS [49,50]. Given this, in the setting of relapsed or refractory neuroblastoma, targeting the RAS-MAPK pathway may provide a therapeutic benefit. Binimetinib, a MEK 1/2 inhibitor, has been shown to inhibit tumor growth and improve survival in mouse models of neuroblastoma [50]. In addition, binimetinib acts synergistically with the CDK4/6 inhibitor, ribociclib, to suppress tumor growth in murine neuroblastoma xenografts [51]. More recent preclinical studies have demonstrated the failure of monotherapy with MEK inhibition in ALK-addicted neuroblastoma due to increased feedback activation of other signaling pathways including PI3K/AKT pathway [52]. There are several MEK inhibitors currently under clinical development. Trametinib, a MEK 1/2 inhibitor, was FDA approved as a single agent (2013) and in combination with the BRAF inhibitor, dabrafenib (2014), for the treatment of patients with unresectable or metastatic melanoma with BRAF V600E or V600K mutations [53,54]. There is currently an ongoing phase 1/2 trial of trametinib alone or in combination with dabrafenib for children with tumors harboring V600 mutations. This study includes an expansion cohort for children with relapsed or refractory neuroblastoma (NCT02124772). In addition, the NEPENTHE study at the Children’s Hospital of Philadelphia is studying the combination of ribociclib and trametinib for patients with relapsed neuroblastoma whose tumors harbor activating mutations in the RAS-MAPK or CDK4/6 pathway (NCT02780128).

## 5. Targeting Telomerase and ALT

Telomeres are regions of repetitive nucleotide sequences found at the end of chromosomes and are crucial for cancer cell survival. Telomeres are shortened during each cell cycle division allowing for cell aging or death in normal cells. Tumor cells however, are able to maintain their telomeres, allowing for infinite proliferative potential. There are two mechanisms in which telomeres can lengthen: through the activity of telomerase or through the alternative lengthening (ALT) pathway, a telomerase independent process that uses homologous recombination [55]. Of late, there has been significant effort invested into understanding the role of telomeres and telomere maintenance in neuroblastoma. Several studies have shown that telomere length correlates with prognosis in neuroblastoma with overall survival only 27% in neuroblastoma cases with telomere lengthening and 89% in cases with telomere shortening (*p* = 0.013) [56].

Telomerase is a reverse transcriptase that involves a catalytic protein subunit called telomerase reverse transcriptase (TERT) encoded by the TERT gene. Telomere maintenance, characterized by telomerase activation, indicates higher invasiveness and poor prognosis in neuroblastoma tumors [57]. TERT mRNA expression in neuroblastoma cases was first detected in 2004; in that cohort, all cases with TERT expression had poor prognosis and were correlated with MYCN expression [58]. Additional studies have demonstrated that down regulation of TERT inhibits proliferation and invasion of neuroblastoma cells and promotes apoptosis [59]. Recent large parallel sequencing studies in neuroblastoma have identified TERT rearrangements, resulting in abnormal telomerase activity, to be involved in telomere maintenance. Whole-genome sequencing of 108 neuroblastoma cases revealed TERT rearrangements in 23% of stage 3 and 4 cases regardless of MYCN amplification or ATRX mutations and also confirmed this to be an independent prognostic factor [60]. An additional study of 217 neuroblastoma cases found TERT rearrangements in 13% of tumors, all but one, stage 4 patients with high-risk disease. Multivariate Cox regression for children >18 months of age demonstrated that MYCN amplification and TERT rearrangement were independent predictors of survival (*p* = 0.0014 and 0.041, respectively) [61]. Given this, inhibition of telomerase is quite appealing and can be accomplished by the G-quadruplex interactive agent, telomestatin. Laboratory-based studies have shown that treatment of neuroblastoma cell lines with telomestatin resulted in dose-dependent cytotoxicity and apoptosis via telomere shortening [62]. Other agents including small-molecule inhibitors, antisense oligonucleotides and immunotherapies have been used to inhibit telomerase function but have yet to be extensively explored in neuroblastoma [63].

The ALT pathway is a telomerase-independent mechanism of telomere length maintenance where telomeric DNA is replicated via homologous recombination using DNA as a template. It is thought that both loss of p53 function, as well as ATRX mutations, permit activation of the ALT pathway [64]. ATRX mutations contribute to ALT activation through the epigenetic regulation of TP53 and, in general, tumors with ATRX mutations have an ALT phenotype. This phenotype includes C-circles (telomeric DNA circles) [65,66,67,68,69]. It has recently been found that the ALT pathway is active in over 50% of neuroblastoma tumors and ALT activity is associated with poor survival. The combination of high TERT expression and ALT activity may represent a novel biomarker of poor prognosis [70]. It has recently been reported that inhibition of the ATR protein kinase disrupts the mechanism of ALT in ALT-positive cancer cells, resulting in cell death [71]. This suggest that ATR inhibitors may be a therapeutic strategy for ALT positive malignancies. These agents are currently being investigated in adults with cancer however, have not yet entered clinical trials in pediatrics.

## 6. TRKA and TRKB Inhibitors

The TRK family of neurotrophin receptors, namely TrkA and TrkB, play a role in the diverse course of and biology of neuroblastoma. Whereas high expression of TrKA is detected in lower risk neuroblastoma prone to spontaneous regression, high expression of TrKB is associated with high-risk disease and poor survival [72,73,74,75]. The pan-Trk inhibitors GNF-4256 and AZD6918 both demonstrated strong activity in pre-clinical models of neuroblastoma, particularly when combined with cytotoxic agents [76,77]. Entrectinib, a tyrosine kinase inhibitor of both ALK and TrkB, also demonstrated potent activity anti-tumor activity when studied in mouse xenografts of neuroblastoma and is currently undergoing evaluation in a phase 1 clinical trial for children with recurrent or refractory solid tumors, with an arm specifically for neuroblastoma (NCT02650401) [78]. Additionally, another TRK inhibitor, larotrectinib (LOXO-101) is under clinical development for both adult and pediatric patients with advanced solid tumors and brain tumors. Expansion cohorts will explore the efficacy of this agent in tumors that harbor alternations in NTRK genes or TRK proteins once the RP2D is determined (NCT02122913). The pediatric MATCH trial is also investigating this agent; however, enrollment is limited to patients with actionable NTRK fusions, and thus is unlikely to enroll neuroblastoma patients (NCT03213704).

## 7. Rho Family of Genes: ROCK Inhibitors

The Rho family of GTPases is fundamental for correct polarization, locomotion, and migration of neural crest cells during embryonal development and is frequently dysregulated in cancer [79,80]. Recently, the Rho-associated kinase has attracted greater interest as a therapeutic target for neuroblastoma after investigators discovered that 39% of high-risk neuroblastoma patients had mutations in genes regulating Rho/Rac signaling. The mutations are associated with activation of the downstream Rho-associated kinases (ROCKSs), with ROCK2 expression correlating with poor prognosis. HA1077, a ROCK1 and ROCK2 inhibitor with a higher preference for ROCK2, was studied in several neuroblastoma cell lines and resulted in significant suppression of cell growth. In mouse models, inhibition of ROCK activity with HA1077 resulted in a 69% reduction in tumor volume compared to controls. Further investigation demonstrated that ROCK2 inhibition promotes MYCN protein degradation [81]. HA1077 is currently being studied in adults for non-malignant conditions but has not yet been investigated for the treatment of malignancies.

## 8. Targeting Epigenetics

With the realization that the epigenetic landscape of cancer cells is quite different from normal cells, an interest in targeting epigenetic changes in neuroblastoma has grown. Epigenetic pathways involving DNA methylation, histone modification, nucleosome remodeling, and non-coding RNAs are linked to oncogenesis [82]. Histone deacetylase (HDAC) inhibitors including panobinostat and vorinostat have been studied extensively in neuroblastoma and have been shown to inhibit tumor growth in pre-clinical models [83,84,85,86]. Clinically, single agent vorinostat does not appear to be effective. Given this, the NANT consortium performed a phase 1 dose escalation study of vorinostat in combination with isotretinoin for patients with relapsed or refractory neuroblastoma. Although no objective responses were seen, 24% of patients had prolonged stable disease and were able to receive anywhere from 11–14 cycles of therapy. When given in an interrupted scheduled, vorinostat doses up to 430 mg/m^2^/day were tolerated [87]. Vorinostat is also known to be a radiosensitizer, and thus was studied in combination with ^131^I-MIBG therapy. Doses of 180 mg/m^2^/dose along with 18 mCi/kg of ^131^I-MIBG were found to be safe and tolerable [88]. A subsequent study through the NANT consortium is comparing ^131^I-MIBG alone to ^131^I-MIBG plus the addition of vincristine and irinotecan or vorinostat (NCT02035137). This trial is ongoing.

Several interesting epigenetics targets are currently gaining more attention in neuroblastoma and will likely undergo further investigation. A recent CRISPR-Cas9 screening of MYCN-amplified neuroblastoma discovered preferential dependency on EZH2. In addition, EZH2 was found to be expressed in high levels in MYCN-amplified neuroblastomas, with MYCN binding at the EZH2 promoter, directly driving expression. Both genetic and chemical inhibition of EZH2 in MYCN-amplified neuroblastoma mouse models resulted in decreased tumor burden. When studied in combination with several small molecules, the EZH2 inhibitor GSK126 was found to be synergistic with HDAC inhibitors [89]. The EZH2 inhibitor, tazemetostat, is currently under evaluation in pediatric patients with INI1-negative tumors or recurrent synovial sarcoma but has not been specifically investigated in patients with neuroblastoma (NCT02601937).

## 9. Inhibiting Anti-Apoptotic Proteins

BCL2 and survivin, two anti-apoptotic proteins, are highly expressed in neuroblastoma wih high level expression correlating with poor prognosis [90,91]. There have been challenges in studying BCL2 inhibitors in preclinical models. In contrast to patient tumors, the majority of neuroblastoma cells lines have low level BCL2, making inhibition ineffective [23,92]. That being said, the BCL2 inhibitors navitoclax and venetoclax were studied in both in vitro and in vivo models of neuroblastoma with encouraging results in MYCN-amplified cell lines and tumors. Investigation into the expression of BCL2 family members that are known to modulate sensitivity to the inhibitors revealed high level expression of NOXA in MYCN amplified cell lines but not in MYCN-wild-type cell lines. It is known that high levels of NOXA expression can confer sensitivity to BCL2 inhibitors and is a likely explanation for the sensitivity of MYC- amplified neuroblastoma cell lines to these agents. Interestingly, when studied in patient derived xenograft models of MYCN-amplified neuroblastoma, venetoclax alone had modest activity however, when combined with the Aurora A kinase inhibitor, alisertib, activity was dramatic with all tumors achieving complete and sustained regression [93]. Single agent venetoclax is currently being evaluated in a phase 1 trial for children with relapsed and refractory malignancies (NCT03236857). Navitoclax is also being studied in children with relapsed or refractory acute lymphoblastic leukemia/lymphoma; however, its use is limited by its side effect of thrombocytopenia (NCT03181126). Inhibition of the anti-apoptotic protein BIRC5, or survivin has also been studied. Inhibition through the use of both locked nucleic acid-based antisense molecule EZN3042 or the small molecule inhibitor YM155 suppressed the growth of neuroblastoma cells in vitro [93,94,95,96]. YM155 is being studied in adult malignancies, but pediatric trials are yet to be activated.

## 10. Inhibition of Autophagy

A challenge with all anti-neoplastic agents is the development of resistance and this is certainly the case with molecularly targeted therapies. A known mechanism of resistance is the induction of autophagy, an important catabolic process that regulates the degradation and recycling of proteins and organelles within a cell. Through this process, cells, including cancer cells, can survive prolonged stress such as exposure to anticancer agents. The induction of autophagy has been demonstrated in certain neuroblastoma cell lines when treated with the ALK inhibitor, entrectinib. The SH-SY5Y cell line, harboring an ALK F1174L mutation, showed less sensitivity to entrectinib than cells lines without this mutation and higher autophagy induction, likely contributing to resistance. When this cell line was co-treated with chloroquine (CQ), an autophagy inhibitor, and entrectinib there was a significant increase in cell death compared to treatment with entrectinib alone. More recently, the receptor tyrosine kinase inhibitors afitinib, sorafenib and TP-0903 were studied both alone and in combination with autophagy blocking agents, CQ and Spautin-1 (SP-1). In these studies, both afitinib and sorafenib caused autophagy induction, whereas TP-0903 only slightly increased the basal level of autophagy in the neuroblastoma cells. When combining the receptor tyrosine kinase inhibitors with either CQ or SP-1, cell death was more pronounced, particularly when treatment with the kinase inhibitor preceded treatment with CQ or SP-1 [97,98]. The use of autophagy inhibition to enhance the efficacy of receptor tyrosine kinase inhibitors for the treatment of neuroblastoma certainly deserves further attention.

## 11. Summary and Conclusions

Neuroblastoma remains one of the challenges within pediatric oncology due to the heterogeneity of the disease and the aggressive nature of disease classified as high-risk. Although the cure rates have increased dramatically over the last decades for patients with high-risk disease, the therapy is quite toxic, resulting in major acute and chronic toxicities. As our understanding of the biology of neuroblastoma advances, so does our ability to discover new druggable targets. The ultimate hope is that the incorporation of rational molecularly guided therapies into the treatment of neuroblastoma will both improve survival and allow for a reduction in other, more toxic, therapies. This review provides an overview of the many molecular aberrations that have been unveiled and the efforts to target these changes in order to improve outcomes. Although much work is still to be done, as we improve our biomarkers and refine risk stratification, we can ultimately optimize drug selection for patients with high-risk neuroblastoma. As the first phase 3 clinical trial for high-risk neuroblastoma incorporating a molecularly targeted therapy is underway, this is truly an exciting time for the development of new agents for neuroblastoma. On the horizon are many possibilities that will hopefully lead to improved outcomes for these patients and time will tell if we can enhance both survival and quality of life through the use of precision medicine. As we move forward with integrating these novel agents into the treatment of high-risk neuroblastoma, it is important to consider the short and long-term impact these agents may have on a young child who still has significant growth potential. The impact on children has yet to be elucidated and may be quite different than the impact on adults. This necessitates a formal investigation as we move forward with these promising therapies.

## Appendix A

**Table A1:** Molecularly targeted agents in clinical development for the treatment of neuroblastoma.

Target	Therapeutic Agents	Study (Reference or Clinical Trial #)
Aurora A	MLN8237 (Alisertib)	Mosse et al. [6], DuBois et al. [7]
ODC1	DFMO	Saulnier Sholler et al. [29] NCT01586260, NCT02030964, NCT02139397
mTOR	Temsirolimus	Bagatell et al. [32], Mody et al. [33]
Pan-PI3K/mTOR	SF1126	NCT02337309
PI3K	Copanlisib	NCT03458728
AKT	Perifosine	Kushner et al. [36]
ALK	Crizotinib	Mosse et al. [39], NCT01606878, NCT03126916
	Ceritinib	NCT01742286, NCT02780128
	Ensartinib	NCT03213652
	Lorlatinib	NCT03107988
MEK 1/2	Trametinib	NCT02124772, NCT02780128
CDK 4/6	Ribociclib	NCT02780128
NTRK	Entrectinib	NCT02650401
	Larotrectinib	NCT02122913
HDAC	Vorinostat	Pinto et al. [87], DuBois et al. [88], NCT02035137
BCL-2	Venetoclax	NCT03236857
BRD4	SF1126	NCT02337309
